# Alterations of lipid metabolism in Wilson disease

**DOI:** 10.1186/1476-511X-10-83

**Published:** 2011-05-19

**Authors:** Jessica Seessle, Annina Gohdes, Daniel Nils Gotthardt, Jan Pfeiffenberger, Nicola Eckert, Wolfgang Stremmel, Ulrike Reuner, Karl Heinz Weiss

**Affiliations:** 1 Department of Gastroenterology, University Hospital Heidelberg, Heidelberg, Germany; 2 Department of Neurology, University Hospital Dresden, Dresden, Germany

**Keywords:** Wilson Disease, lipids, cholesterol, triglycerides, liver disease

## Abstract

**Introduction:**

Wilson disease (WD) is an inherited disorder of human copper metabolism, characterised by accumulation of copper predominantly in the liver and brain, leading to severe hepatic and neurological disease. Interesting findings in animal models of WD (Atp7b^-/- ^and LEC rats) showed altered lipid metabolism with a decrease in the amount of triglycerides and cholesterol in the serum. However, serum lipid profile has not been investigated in large human WD patient cohorts to date.

**Patients and Methods:**

This cohort study involved 251 patients examined at the Heidelberg and Dresden (Germany) University Hospitals. Patients were analysed on routine follow-up examinations for serum lipid profile, including triglycerides, cholesterol, high density lipoprotein (HDL) and low density lipoprotein (LDL). Data on these parameters at time of diagnosis were retrieved by chart review where available. For statistical testing, patients were subgrouped by sex, manifestation (hepatic, neurological, mixed and asymptomatic) and treatment (D-penicillamine, trientine, zinc or combination).

**Results:**

A significant difference in total serum cholesterol was found in patients with hepatic symptoms, which diminished under therapy. No alterations were observed for HDL, LDL and triglycerides.

**Conclusion:**

Contradictory to previous reports using WD animal models (Atp7b^-/- ^and LEC rats), the most obvious alteration in our cohort was a lower serum cholesterol level in hepatic-affected patients, which might be related to liver injury. Our data suggested unimpaired cholesterol metabolism in Wilson disease under therapy, independent of the applied medical treatment.

## Background

Wilson disease (WD) is an autosomal recessive inherited disorder of copper metabolism caused by mutations of the hepatic copper transporting ATPase ATP7B, which facilitates biliary copper excretion. Thus ATP7B dysfunction leads to toxic copper accumulation in the liver and other tissues, most notably the central nervous system [1,2]. The genetic background of the disease is highly variable with more than 300 ATP7B mutations known so far [3-5]. Liver injury is the most common manifestation of Wilson disease and it ranges from steatosis hepatis to irreversible liver cirrhosis and fulminant liver failure [6,7] with a variable age at onset [8]. The liver is not only responsible for maintenance of copper homoestasis but plays a central role in lipid transport and metabolism. Thus liver disease can be associated with profound and characteristic changes in lipoprotein composition, metabolism and transport [9]. However, alterations of lipid metabolism in humans with copper overload disease have not been investigated in larger cohorts so far.

Here, some data are available for rodent models of WD, the Atp7b^-/- ^mouse and the LEC rat. Atp7b knock-out mice exhibit down-regulated lipid metabolism [10]. A significant decrease in the amount of triglycerides and cholesterol was detected, whereas LDL cholesterol remained unchanged [10]. Very low density lipoprotein (VLDL) fraction was the most affected, showing a 3.6-fold reduction in cholesterol concentration. Analysis of liver tissue revealed that cholesterol was indeed markedly decreased [10]. In contrast, Levy et al. showed an increased content of triglycerides, free cholesterol and cholesteryl ester in the liver of LEC rats. This steatosis was associated with hypotriglyceridemia, hypocholesterinemia and abnormalities in both circulating lipoprotein composition and size [11]. In both animal models of Atp7b^-/- ^mice and LEC rats, there is a significant effect of accumulated copper on hepatic HMG-CoA reductase (the rate limiting enzyme in *de novo *cholesterol biosynthesis). HMG-CoA reductase mRNA levels were most significantly down-regulated in Atp7b^-/- ^mice [10] while the activity of the enzyme was decreased in LEC rats [11].

Several other animal studies support the idea that lipid and copper metabolism are linked. For example, copper deficient rats showed elevated total cholesterol and triglyceride plasma levels and a significant decrease in hepatic total cholesterol [12]. HMG-CoA reductase was elevated two-fold with copper deficiency [12,13]. In male Wistar rats, Galhardi et al. showed reduced cholesterol and triglycerides, total cholesterol and LDL in the serum after dietary copper supplementation. However, there was no significant change in final body weight and high-density lipoprotein [13,14].

There are limited data regarding Wilson disease and the alteration of lipid metabolism in humans. Brewer et al. demonstrated in eleven female and thirteen male WD patients that zinc treatment reduced cholesterol levels by about 10% in both sexes and reduced HDL cholesterol level by about 20% in male patients [15]. Rodo et al. investigated 45 WD individuals treated with D-pencillamine or zinc sulphate and demonstrated significantly lower levels of cholesterol, LDL cholesterol and Vitamin E compared with untreated healthy controls. No differences were seen in triglyceride levels between both groups [16].

These findings were in line with other smaller series in healthy male individuals that evaluated the role of zinc in lipid reducing therapy [17-19]. Black et al. examined the effect of oral zinc supplementation for twelve weeks in thirty one healthy men (9 subjects in the placebo group, 13 in the 50mg Zn/d group and 9 in the 75 mg Zn/d group) in a double blinded study [19]. No changes in total serum cholesterol, LDL, VLDL and triglycerides were observed. Hooper et al. showed a 25% decrease below baseline values of HDL in 12 healthy men after zinc supplementation [17]. Total cholesterol, triglycerides and LDL cholesterol levels did not change. Similar data were revealed by Chandra in 11 healthy men: HDL concentration decreased significantly and LDL increased slightly after ingestion of elemental zinc [18]. The mechanism of these effects of zinc on cholesterol metabolism is currently unclear. The aim of the current study was to assess lipid metabolism parameters in patients with Wilson disease at initial presentation and in course under medical therapy.

## Results

### Findings at initial presentation

In untreated Wilson disease, median serum cholesterol and triglycerides presented within the normal range. A comparison between male and female patients showed no significant differences concerning serum lipids (Table 1).

**Table 1:** A comparison between treatment naive male and female patients showed no significant differences concerning serum lipids.

	**n** **m:f**	**Limits of normal**	**Total**	**Male**	**Female**	**p-value**
	
**ALT**	139 55:84	f: -35 U/l m: -50 U/l	**62.3** (7-3743)	**95** (7-3743)	**54** (7-472)	**0.032**
	
**AST**	135 55:80	f: -35 U/l m: -50 U/l	51.9 (6-2106)	52 (6-2106)	50 (13-323)	0.485
	
**Bilirubin**	122 44:78	< 1,0 mg/dl	0.9 (0.1-39)	0.9 (0.2-23)	0.9 (0.1-39)	0.974
	
**Albumin**	103 37:66	30-50 g/l	43 (23-57)	43.9 (25-55)	42.6 (23-57)	0.179
	
**TG**	64 26:38	< 150 mg/dl	114 (33-888)	149.5 (59-313)	106.5 (33-888)	0.061
	
**Cholesterol**	70 27:43	< 200 mg/dl	175.5 (66-319)	186 (93-319)	170 (66-294)	0.436
	
**HDL**	14 7:7	f: >50 mg/dl m: >40 mg/dl	56.5 (42-77)	53 (42-67)	57 (48-77)	0.179
	
**LDL**	12 7:5	< 160 mg/dl	96 (63-218)	97 (63-218)	95 (63-166)	0.935
	
**BMI**	26 12:14	18.5-25 kg/m²	22.1 (14.8-30.8)	22.9 (15.3-27.7)	21.2 (14.8-30.8)	0.877

No differences were demonstrated between both sexes in the lipid metabolism parameters.

**Figure 1:**
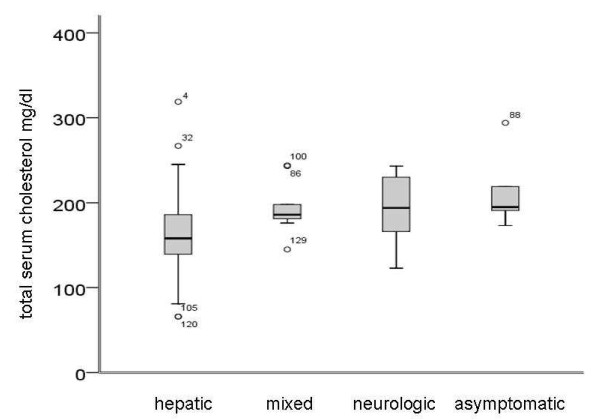
**Serum cholesterol subgrouped by presentation at initial presentation (n = 70)**. Median total serum cholesterol was reduced in hepatic patients (*p *= 0.004).

For further analysis, patients were subgrouped by presentation (neurologic, hepatic, mixed and asymptomatic). As expected, patients with hepatic manifestation showed higher levels of liver function tests namely alanine aminotransferase (ALT) and aspartate aminotransferase (AST), bilirubin and lower levels of serum albumin. Total cholesterol was lower in patients with hepatic symptoms (Figure 1) while no alterations in the other analysed parameters of lipid metabolism were noted (Table 2).

**Table 2:** Baseline parameters of treatment naive patients subgrouped by different manifestation revealed a significant decrease in cholesterol in hepatic patients.

	**n**	**Limits of normal**	**Hepatic**	**Neurological**	**Mixed**	**Asymptomatic**	**p-value**
	
**ALT**	139	f: -35 U/l m: -50 U/l	**101.9** (7-3743)	**22** (10-140)	**28.2 **(17-272)	**20** (7-168)	**>0.001**
	
**AST**	135	f: -35 U/l m: -50 U/l	**76.9** (13-2106)	**21.2** (6-58)	**32** (19-108)	**26.4** (11-102)	**>0.001**
	
**Bilirubin**	122	< 1.0 mg/dl	**1.1** (0.2-39)	**0.9** (0.1-3.8)	**0.6** (0.2-1.4)	**0.5** (0.2-0.9)	**0.009**
	
**Albumin**	103	30-50 g/l	**41** (23-55)	**45.2** (38-52)	**43** (35-57)	**46.3** (35-52)	**0.001**
	
**TG**	64	< 150 mg/dl	110 (33-888)	120 (63-200)	124 (40-249)	60 (59-100)	0.300
	
**Cholesterol**	70	< 200 mg/dl	**158** (66-319)	**194** (123-243)	**186** (145-244)	**195** (173-294)	**0.004**
	
**HDL**	14	>50 mg/dl	57 (42-76)	53 (48-77)	60 (56-64)	-	0.923
	
**LDL**	12	< 160 mg/dl	96 (63-218)	161 (156-166)	88 (81-95)	-	0.207
	
**BMI**	26	18.5-25 kg/m²	20.6 (14.9-24.5)	26.2 (20.3-30.8)	23.1 (14.8-28.6)	19.7 (19.7-19.7)	0.126

Median serum cholesterol and triglycerides presented within normal range.

### Course under medical treatment

Under at least 2 years of therapy, the differences within LFTs (ALT, AST) diminished, reflecting the hepatic response to medical treatment. With improvement of liver function, parameters of lipid metabolism - in particular cholesterol levels - were comparable between patients, independent of initial presentation (Table 3). Moreover, it was noticeable that lipoprotein a, an independent risk factor for atherosclerosis, was significantly decreased in those individuals with hepatic and mixed presentation.

**Table 3:** Parameters of lipid metabolism of Wilson disease patients under therapy.

	**n**	**Limits of normal**	**Hepatic**	**Neurological**	**Mixed**	**Asymptomatic**	**p-value**
	
**ALT**	249	f: -35 U/l m: -50 U/l	**41** (10-3743)	**25** (11-70)	**30** (13-99)	**24.5** (8-78)	**<0.001**
	
**AST**	250	f: -35 U/l m: -50 U/l	**29** (12-2106)	**23** (10-102)	**27.9 **(12-79)	**20** (11-77)	**<0.001**
	
**Bilirubin**	239	< 1.0 mg/dl	**0.7** (0.2-23.0)	**0.6** (0.2-2.7)	**0.7** (0.2-4.5)	**0.6** (0.3-1.9)	**0.001**
	
**Albumin**	231	30-50 g/l	44 (21-55)	44.4 (28-56)	44 (30-73)	45.6 (37-54)	0.277
	
**TG**	247	< 150 mg/dl	100 (28-888)	95 (29-438)	106.5 (12-221)	93 (29-285)	0.588
	
**Cholesterol**	248	< 200 mg/dl	179.5 (90-319)	182 (120-365)	183 (113-273)	188.5 (103-265)	0.780
	
**HDL**	124	>50 mg/dl	55 (27-113)	53 (32-103)	57.5 (40-81)	57 (36-84)	0.588
	
**LDL**	115	< 160 mg/dl	108 (43-218)	96 (63-229)	98.5 (63-159)	113.5 (74-156)	0.781
	
**Lp(a)**	61	< 25 mg/dl	**4.8** (5-52.5)	**21.4** (4.8-48.5)	**4.9** (4.8-55.4)	**20.5** (4.8-38.9)	**0.016**
	
**BMI**	166	18.5-25 kg/m²	22.8 (14.2-41.1)	22.1 (17.7-33.7)	23.8 (15.1-32.5)	22.1 (18.7-28.1)	0.839

With improvement of liver function, parameters of lipid metabolism were comparable between patients, independent of initial presentation.

To evaluate the influence of different treatment regimens, data for patients under therapy were analysed by treatment (Table 4). For cholesterol and serum lipids, no statistically significant differences were observed between the groups. Under zinc and combination treatment, significantly higher levels of lipase and amylase, known side effects of the medication, were observed.

**Table 4:** In patients subgrouped by different treatment regimens, no differences in lipid metabolism parameters were demonstrated, even under zinc monotherapy.

	**n**	**Limits of normal**	**DPA**	**Trientine**	**Zinc**	**Combination**	**p-value**
	
**ALT**	249	f: -35 U/l m: -50 U/l	36 (8-3743)	30 (10-398)	34.5 (14-162)	34 (12-100)	0.133
	
**AST**	250	f: -35 U/l m: -50 U/l	28 (11-2106)	27 (12-167)	25 (10-110)	24 (12-79)	0.775
	
**Bilirubin**	239	< 1.0 mg/dl	0.7 (0,3-23)	0.6 (0.2-4.5)	0.6 (0.2-10)	0.8 (0.3-1.8)	0.306
	
**Albumin**	231	30-50 g/l	**44.2** (28-54)	**43** (21-55)	**46** (24-73)	**44.4** (30-56)	**0.033**
	
**TG**	247	< 150 mg/dl	106 (24-888)	97 (29-287)	94 (12-438)	87 (44-178)	0.505
	
**Cholesterol**	248	< 200 mg/dl	179 (90-288)	185.5 (107-365)	187 (122-340)	172 (137-259)	0.780
	
**HDL**	124	f: >50 mg/dl m: >40 mg/dl	56 (27-103)	59.5 (39-113)	54 (39-100)	48.5 (32-90)	0.467
	
**LDL**	115	< 160 mg/dl	102.5 (43-204)	98 (52-218)	113 (51-229)	94.5 (64-151)	0.324
	
**BMI**	166	18.5-25 kg/m²	23.7 (18.1-33.7)	22.5 (14.2-41.1)	21.9 (15.1-34.1)	25.2 (18.5-29)	0.060

## Discussion

The liver is a central organ in lipid transport and metabolism. It is well known that liver diseases are associated with profound and characteristic changes in lipoprotein composition and metabolism [9]. Only limited data are currently available on the detailed lipoprotein alterations in Wilson disease. The aim of the current study was to investigate alterations in lipid metabolism parameters in WD patients.

The study examined 251 Wilson disease patients. Our expectation with regard to animal models was to show altered parameters of lipid metabolism. Despite the fact that differences in VLDL were a major finding in the rodent studies [10] we focused on the lipid parameters assessed in clinical routine to follow WD patients. This represents a limitation of our study.

While Atp7b knockout mice showed down-regulated lipid metabolism with a significant decrease in the amount of triglycerides and cholesterol [11,13], in our human cohort median serum cholesterol and triglycerides presented within the normal range. A lower median cholesterol was only evident in the subgroup of patients with hepatic symptoms. Whether this finding is a specific effect of the copper overload or just an epiphenomenon of liver injury remains open, as previous studies observed a clear trend towards low levels of total cholesterol in patients chronic liver disease [9]. All other analysed parameters of lipid metabolism were inconspicuous even in the hepatic patients. In further course under therapy the finding of lower cholesterol in patients with hepatic presentation was no longer evident. After two years of treatment the analyzed lipid metabolism parameters were comparable between patients, independent of the initial presentation.

To evaluate the influence of different treatment regimens, data were also analysed by treatment. For cholesterol and serum lipids, no statistically significant differences were observed between different medical regimes. Thus, in our cohort we could not verify the previous observation of reduced cholesterol levels under zinc therapy [15,16].

In summary, our study at least in part contradicts the findings obtained in Wilson disease animal models. The most obvious alteration was a lower serum cholesterol level in hepatic- affected patients, which might be related to liver injury. Our data suggested an unimpaired cholesterol metabolism in Wilson disease under therapy, regardless of the applied treatment regimen.

## Material and methods

### Patients

This cohort study involved 251 patients with an established diagnosis of Wilson disease examined at the University Hospitals of Heidelberg and Dresden between 1998 and 2009. The study protocol was a priori approved by the ethical committee of the University of Heidelberg.

The diagnosis of this disease was based on the critieria of the 8^th ^International Conference of Wilson disease and Menkes disease [20]. Patients with uncertain diagnosis were excluded as described previously [21]. In terms of a cross sectional study, all patients were analysed on routine follow-up examinations for serum lipid profile, including triglycerides, cholesterol, HDL and LDL. Data on serum lipids at time of diagnosis were retrieved by chart review and were not available for all cases. For statistical testing, they were subgrouped by treatment (d-penicillamine, trientine, zinc and combination), sex and manifestation (hepatic, neurological, mixed and asymptomatic). P-values were retrieved using Mann-Whitney or Kruskal-Wallis test.

### Statistical methods

A p-value less than 0.05 was considered significant. Calculation was carried out using SPSS™ for Windows™ Software V15.0.

## Abbreviations

ALT: alanine aminotransferase; AST: aspartate aminotransferase; BMI: body mass index; Cp: ceruloplasmin; DPA: D-penicillamine; HDL: high-density lipoproteins; LEC: long evans chinnamon; LDL: low-density lipoproteins; LFT: liver function test; Lp(a): lipoprotein a; TG: triglycerides; VLDL: very low-density lipoproteins; WD: Wilson disease

## Competing interests

The authors declare that they have no competing interests.

## Authors' contributions

KHW, JS were involved in the study concept and design; acquisition of data; analysis and interpretation of data; statistical analysis; drafting of the manuscript; study supervision; critical revision of the manuscript for important intellectual content/AG, NE were involved in the acquisition of data/DNG, JP, WS, UR were involved in the acquisition of data; critical revision of the manuscript for important intellectual content; statistical analysis.

All authors read and approved the final manuscript.
